# Supplementary material for: The structure and catalytic mechanism of a pseudoknot-containing hammerhead ribozyme
Source: Nat Commun. 2024 Aug 5;15:6628. doi: 10.1038/s41467-024-50892-y (PMC11300833; doi:10.1038/s41467-024-50892-y)
Supplement: Supplementary file 1 — Supplementary Information [file 41467_2024_50892_MOESM1_ESM.pdf]

The structure and catalytic mechanism of a pseudoknot-containing hammerhead ribozyme

Xuelin Zhan, Timothy J. Wilson, Zhenzhen Li, Jingjing Zhang, Yili Yang, David M. J. Lilley and Yijin Liu

SUPPLEMENTARY MATERIAL

SUPPLEMENTARY FIGURES

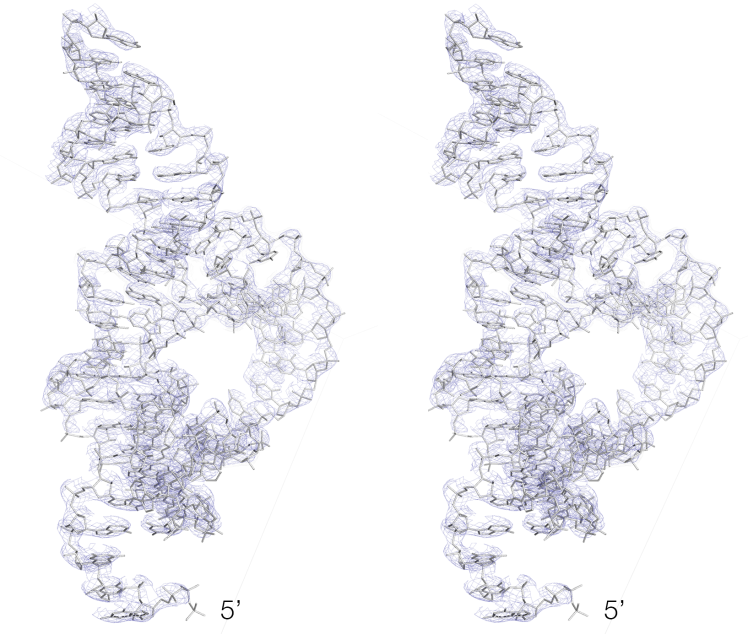

Supplementary Figure S1. Parallel-eye view of the crystal structure of the PK hammerhead ribozyme showing the 2$\mathbf{F_o}$-$\mathbf{F_c}$ electron density map contoured at 2.5 σ.

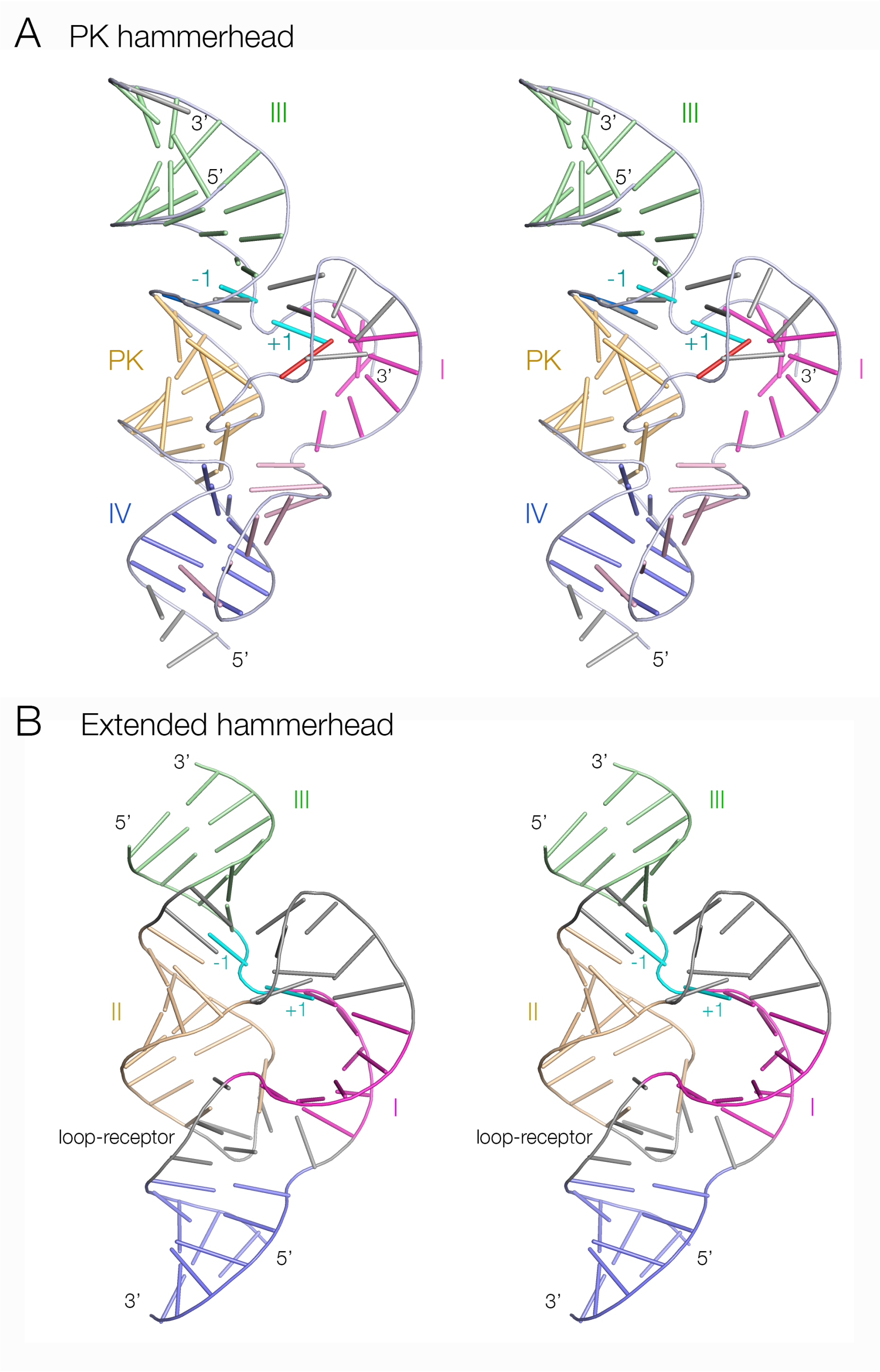

Supplementary Figure S2. Comparison of the overall structures of the extended hammerhead and PK hammerhead ribozyme structures. Both structures shown as parallel-eye stereoscopic views. **A.** The PK hammerhead ribozyme determined in this work. **B.** The extended hammerhead ribozyme structure of Martick and Scott (PDB ID 3ZD5).

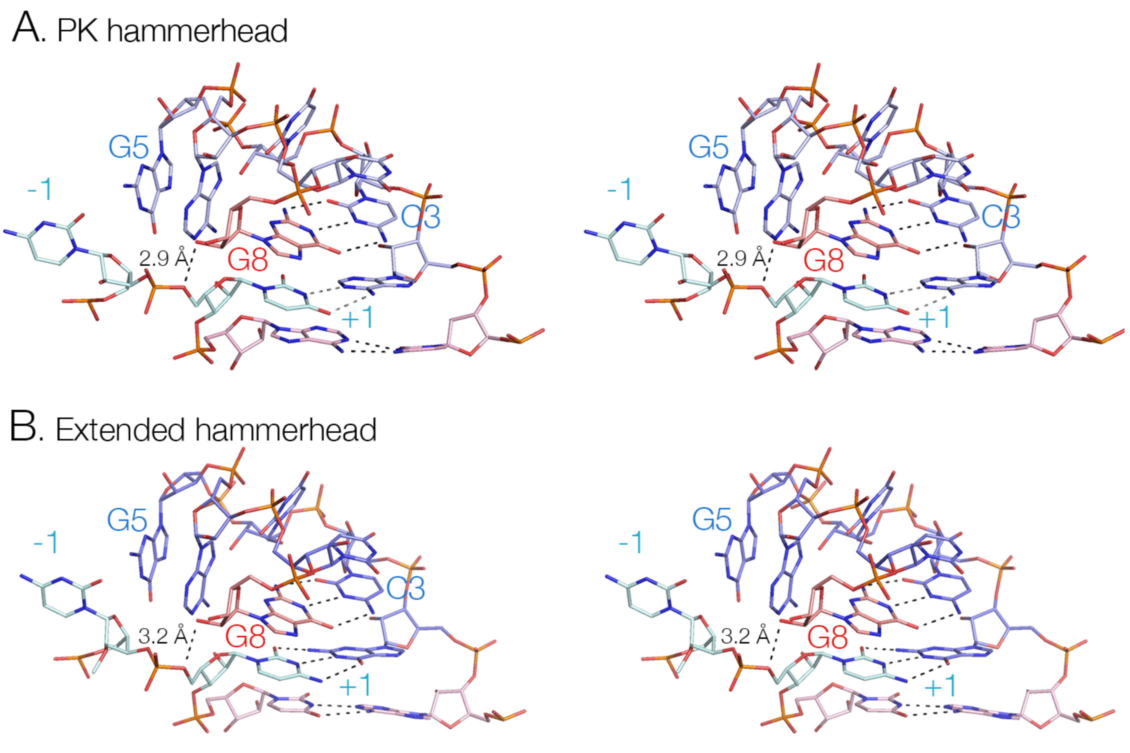

Supplementary Figure S3. Comparison of the loop structures in the PK hammerhead and the extended hammerhead ribozymes. Parallel-eye stereoscopic views of **A**. the PK hammerhead ribozyme loop, and **B**. the extended hammerhead ribozyme.

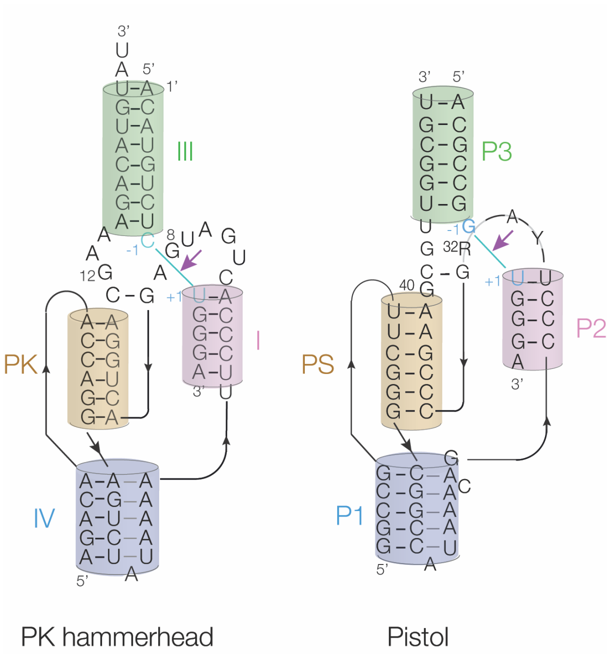

Supplementary Figure S4. Schematic drawings of the secondary structures of the PK hammerhead and pistol ribozymes. This shows that the connectivity of the helices in the two ribozymes are essentially identical. The arrows indicate the position of ribozyme cleavage.

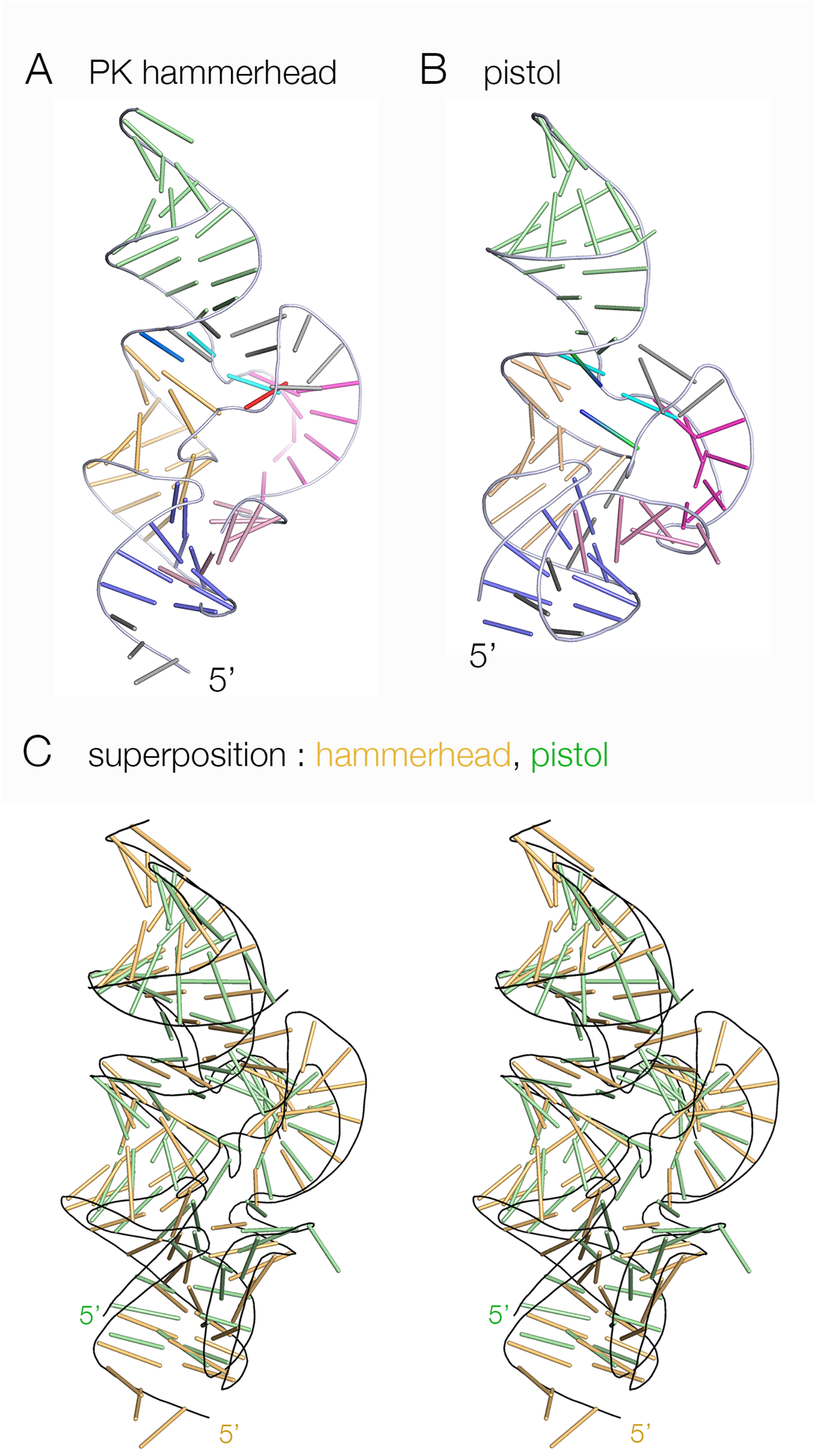

Supplementary Figure S5. Comparison of the overall structures on the PK hammerhead and pistol ribozymes. **A**. The structure of the PK hammerhead ribozyme. **B**. The structure of the pistol ribozyme in approximately the same view, **C**. Parallel-eye stereoscopic view of a super-position of the PK hammerhead structure (orange) with the pistol structure (green). The structures were superimposed using all atoms (1100 atoms), with an RMSD = 3.96 Å. The pistol ribozyme structure used was PDB 6R47 (1).

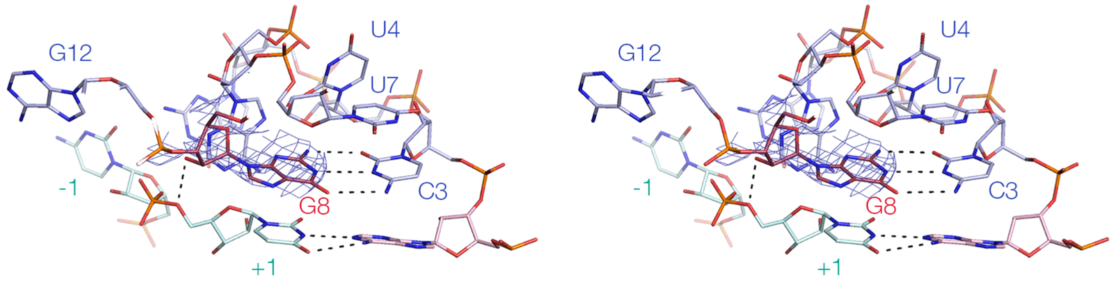

Supplementary Figure S6. Parallel-eye stereoscopic view of the loop of the PK hammerhead ribozyme with the electron density map shown for the general acid G8. The 2$\mathbf{F_o}$-$\mathbf{F_c}$ electron density map is shown contoured at 2.5 σ.

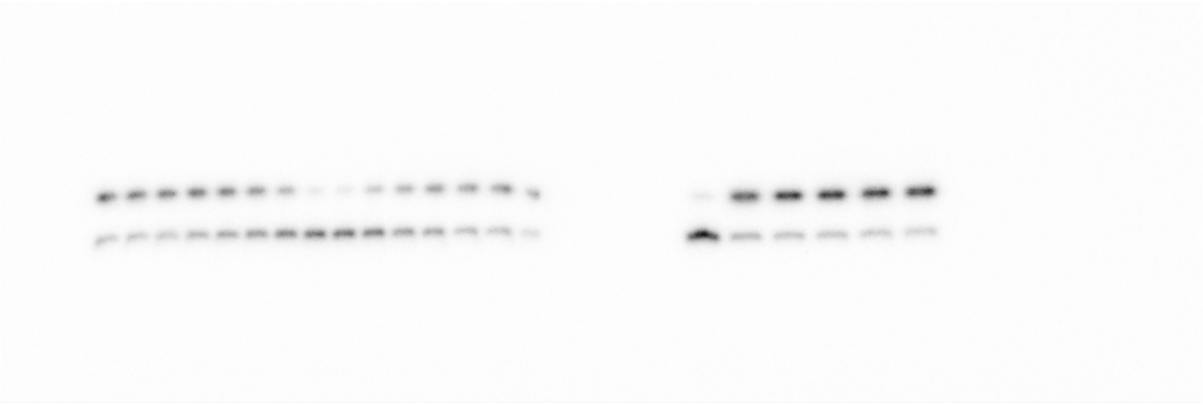

Supplementary Figure S7. Uncropped gel showing reaction progress for unmodified (with and without Mg^{2+}) and dG8 (with Mg^{2+}) PK hammerhead ribozymes (as presented in Figure 6).

SUPPLEMENTARY TABLES

Crystal	PK-HH	PK-HH (Br)
**Data collection**		
Wavelength/ Å	0.97918	0.9199
	16.1 - 2.89	62.87 - 3.5
Resolution range/ Å	(2.993 - 2.89)	(3.625 - 3.5)
Space group	P 61 2 2	P 61 2 2
Unit cell	83.58, 83.58, 258.79Å	82.99, 82.99, 259.44 Å
	90°, 90°, 120°	90°, 90°, 120°
Total reflections	478822 (49468)	272943 (27572)
Unique reflections	12665 (1226)	7285 (706)
Multiplicity	37.8 (40.3)	37.5 (39.1)
Completeness (%)	98.38 (98.45)	99.51 (98.87)
Mean I/sigma(I)	18.05 (1.35)	22.87 (3.14)
Wilson B-factor	78.25	113.68
R-merge	0.1874 (3.004)	0.125 (0.5353)
R-meas	0.1899 (3.042)	0.1268 (0.5423)
CC1/2	1.000 (0.866)	1 (0.994)
Anomalous slope		0.408
Refinement		
R-work	0.1953 (0.4132)	
R-free	0.2097 (0.4478)	
CC(work)	0.962 (0.630)	
Number of atoms		
non-hydrogen	1549	
macromolecules	1517	
ligands	32	
RMSD		
RMS(bonds) /Å	0.009	
RMS(angles) / °	1.87	
Average B-factor	81.52	
PDB:	8YDC	

Statistics for the highest resolution shell are in parentheses. RMSD = root mean square deviation

Supplementary Table 1. Detailed information on crystal data collection and refinement statistics.

	standard nomenc.	PDB file
C	-1	9
U	+1	10
A	2.1	28
C	3	29
U	4	30
G	5	31
A	6	32
U	7	33
G	8	34
A	9	35
G	10.1	36
C	11.1	43
G	12	44

Supplementary Table 2. Nucleotide nomenclature in the core of the PK hammerhead ribozyme. This corelates the standard nomenclature for nucleotide positions (2) and the numbering in the PDB file of the structure.

References for Supplementary Information

1. Wilson, T.J., Liu, Y., Li, N.S., Dai, Q., Piccirilli, J.A. and Lilley, D.M.J. (2019) Comparison of the structures and mechanisms of the pistol and hammerhead ribozymes. *J Amer. Chem. Soc.*, **141**, 7865-7875.
2. Hertel, K.J., Pardi, A., Uhlenbeck, O.C., Koizumi, M., Ohtsuka, E., Uesugi, S., Cedergren, R., Eckstein, F., Gerlach, W.L., Hodgson, R. *et al.* (1992) Numbering system for the hammerhead. *Nucleic Acids Res.*, **20**, 3252.
